# Calreticulin mediates an invasive breast cancer phenotype through the transcriptional dysregulation of p53 and MAPK pathways

**DOI:** 10.1186/s12935-016-0329-y

**Published:** 2016-07-13

**Authors:** Mohammadreza Zamanian, Lama Abdel Qader Hamadneh, Abhi Veerakumarasivam, Sabariah Abdul Rahman, Shamarina Shohaimi, Rozita Rosli

**Affiliations:** Department of Genetics, Reproductive Biomedicine Research Center, Royan Institute for Reproductive Biomedicine, ACECR, Tehran, Iran; Medical Genetics Laboratory, Faculty of Medicine and Health Sciences, Universiti Putra Malaysia, 43400 Serdang, Selangor Darul Ehsan Malaysia; Department of Pharmacy, Faculty of Pharmacy, Al-Zaytoonah University of Jordan, Amman, 11733 Jordan; Perdana University Graduate School of Medicine, Perdana University, 43400 Serdang, Selangor Darul Ehsan Malaysia; Cluster of Medical Laboratory Sciences, Faculty of Medicine, Universiti Teknologi MARA, Selayang Campus, 68100 Batu Caves, Selangor Malaysia; Department of Biology, Faculty of Science, Universiti Putra Malaysia, 43400 Serdang, Selangor Malaysia; UPM-MAKNA Cancer Research Laboratory, Institute of Bioscience, Universiti Putra Malaysia, 43400 Serdang, Selangor Malaysia

**Keywords:** Calreticulin, Breast cancer, Invasion, p53, MAPK

## Abstract

**Background:**

The introduction of effective novel biomarkers of invasion and metastasis is integral for the advancement of breast cancer management. The present study focused on the identification and evaluation of calreticulin (CRT) as a potential biomarker for breast cancer invasion.

**Methods:**

Two-dimensional gel protein electrophoresis and MALDI-TOF were utilized in the analysis of fresh-frozen invasive intra-ductal carcinoma specimens. Calreticulin-associated expression was analyzed using immunohistochemistry of FFPE non-malignant/malignant breast specimens. A *CRT*-knockdown model of MCF7 cell line was developed using siRNA and the *CRT* genotype/phenotype correlations based on migration and trans-well invasion assays were determined. Finally, microarray-based global gene expression profiling was conducted to elucidate the possible calreticulin pro-invasive regulatory pathways.

**Results:**

Two-dimensional gel protein electrophoresis and MALDI-TOF analysis showed upregulation of calreticulin expression in tumor tissues as compared to the normal adjacent tissues. Meta-analysis of the immunohistochemical results confirmed significantly higher expression of calreticulin (p < 0.05) in the stromal compartments of malignant tissues as compared to non-malignant tissues. Migration and transwell invasion assays showed significant loss in the migratory and invasive potential of *CRT*-knockdown cells (p < 0.05). Global gene expression profiling successfully identified various putative gene networks such as p53 and MAPK pathways that are involved in calreticulin breast cancer signaling.

**Conclusion:**

Besides confirming calreticulin overexpression in invasive breast cancer tissues, this study reveals a calreticulin-dependent pro-invasive potential and suggests possible contributing pathways. Defining the mechanistic role of invasion and characterizing the possible calreticulin-dependent molecular targets will be the focus of future work.

**Electronic supplementary material:**

The online version of this article (doi:10.1186/s12935-016-0329-y) contains supplementary material, which is available to authorized users.

## Background

Breast cancer is a major health problem for women as it is diagnosed at least a million times every year worldwide [1]. In the United States, it is the second leading cause of cancer deaths and the most common cancer among women [2]. In Asian countries, the peak age for the disease is between 40 and 50 with an increasing trend in incidence and associated mortality [3]. The ability to accurately predict the presence of distant metastasis; the direct consequence of the invasive ability of cancer cells significantly aids in the determination of effective patient management strategies [4].

Calreticulin (CRT) is an important endoplasmic reticulum (ER) protein with critical functions inside and outside the ER [5]. Protein chaperoning and calcium homeostasis are the two key functions of calreticulin inside the lumen of the ER [6]. Outside the ER, calreticulin is involved in cell adhesion [7], modification of gene expression [8], and immune response [9]. Calreticulin is also reported to be correlated with cellular proliferation and metaplastic events [8]. In addition, calreticulin has also been linked to malignant states [10], and as a major calcium homeostasis contributor, it plays a role in cancer invasion and metastasis [11]. Recent studies have focused on revealing an association between the presence of calreticulin and the initiation or progression of various malignant transformations [12]. In brief, the tumor-promoting effects of calreticulin or its overexpression has been reported in ductal carcinoma of the breast [13], bladder cancer [14], and prostatic adenocarcinoma [15]. Furthermore, hepatocellular carcinoma [16], pancreatic malignancies [17], esophageal cancer [18], gastric cancer [19], colon cancer [20], melanoma [21], and leukemia [22] have also been reported to be potentially correlated with calreticulin activity.

A higher expression of calreticulin protein in breast ductal adenocarcinomas has been previously reported [13], which possibly indicates to a correlation between the presence of calreticulin and the initiation and/or progression of cancer. Calreticulin overexpression was also found to be associated with a higher risk of developing invasion and metastasis in breast cancer patients [23]. In addition, calreticulin has been suggested to be a potential biomarker for breast cancer prognosis as its expression was associated with more advanced tumors [24].

Taken together, calreticulin is believed to be involved in the initiation and progression of various cancers. In breast cancer, calreticulin expression has been correlated with more advanced disease and a higher chance for the development of distant metastasis. As the mechanisms and the pathways through which calreticulin may contribute to breast cancer invasion and metastasis have not been determined, we first tried to clarify the importance of calreticulin stromal expression in conferring a more invasive phenotype to breast cancer and to subsequently propose possible mechanisms or pathways for calreticulin pro-invasive effects. The proteomic component of the present study revealed calreticulin as differentially expressed in various invasive states of breast cancer. Then we focused on evaluating the possible correlations between calreticulin expression and the conferment of an invasive phenotype in breast cancer cells as well as clarifying the related mechanisms. We aimed to probe the patterns of differential expression of calreticulin in a range of human breast tissues including non-malignant and malignant lesions. Subsequently, we established the role of *CRT* in conferring an invasive phenotype in breast cancer cells through siRNA gene knockdown and whole genome expression profiling.

## Results

**Fig. 1:**
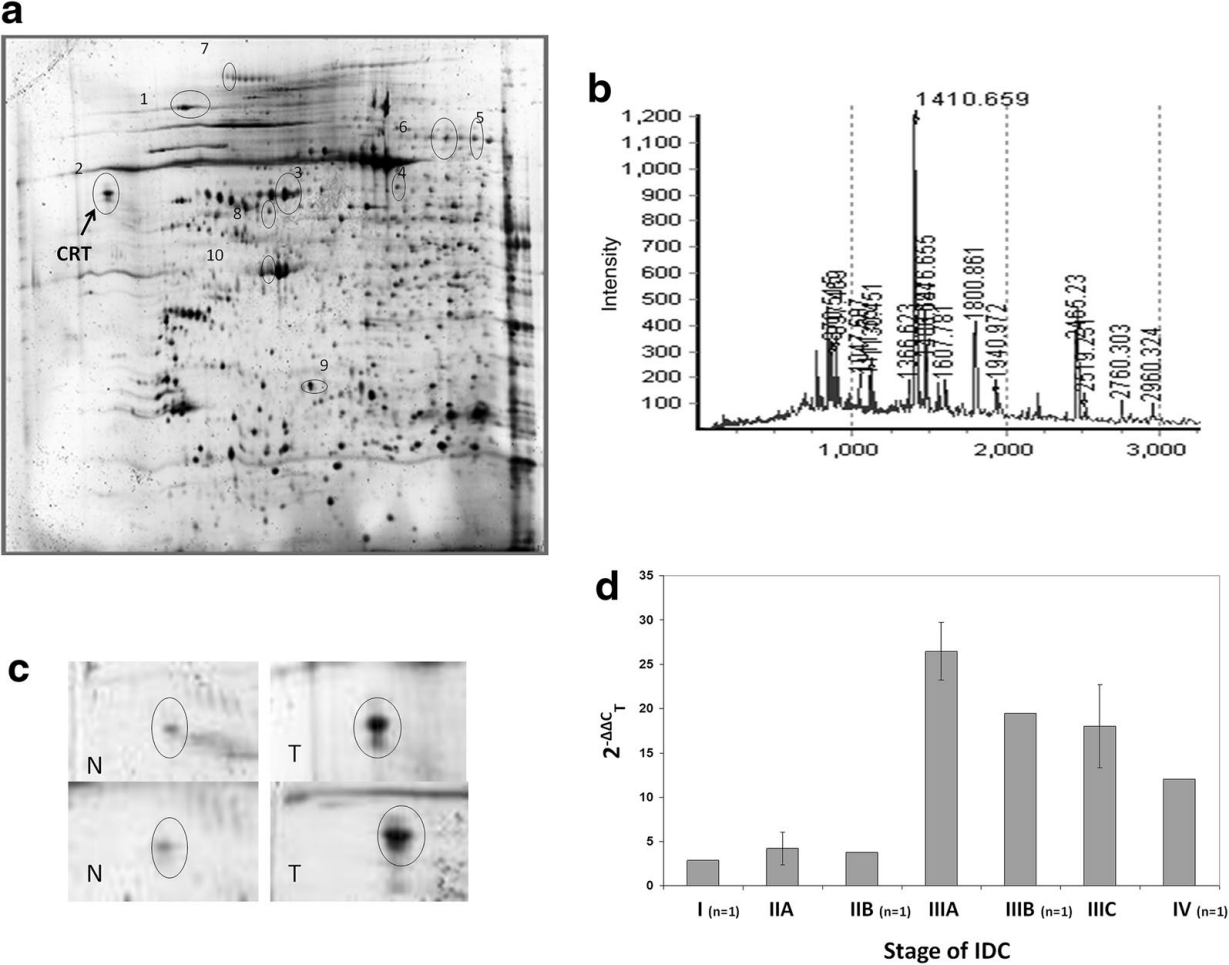
Two-dimensional gel electrophoresis and MALDI-TOF mass spectra analysis reveal calreticulin as over expressed in breast cancer tissues. **a** A representative 2D gel electrophoresis map for the tumor tissue from a stage III sample after running on 17 cm pH 4–7 IPG strips. Spots 1 through 9 were excised and the protein identification was determined by MALDI-TOF analysis, in which spots 1–5 and spot 9 were over-expressed in the tumor tissue, while spots 6–8 were down-regulated. **b** MALDI-TOF mass spectrum of the tryptic peptides corresponding to calreticulin. **c** Expression of calreticulin in different intra ductal carcinoma (IDC) samples (*T* tumoral tissue, *N* normal adjacent tissue). **d** Graph represents the *CRT *expression fold change across different stages of infiltrating ductal breast carcinoma specimens

Two-dimensional gel electrophoresis protein maps in the range of pH 4–7 were obtained in duplicates and protein spots that were differentially expressed between tumor and adjacent normal epithelial tissues were selected. Figure 1a shows a representative gel where the protein spots are circled. Spots that were differentially expressed across different samples were excised and sent for MALDI-TOF analysis. Figure 1b represents the MALDI-TOF spectrogram for calreticulin tryptic peptides while all of the correlated identified spots are also listed in Additional file 1: Table S1. The results revealed that calreticulin is overexpressed in the tumoral regions of the tissue samples (spot 2 in Fig. 1a). Figure 1c represents calreticulin expression in different intra ductal carcinoma (IDC) samples including the tumoral and normal adjacent tissues. By using qPCR, *CRT* expression was confirmed to be higher in stages III and IV of breast cancer as compared to stage I and II tumors (Fig. 1d). Results from protein profiling revealed differential calreticulin expression patterns between tumoral and normal adjacent tissues of breast cancer specimens.

**Fig. 2:**
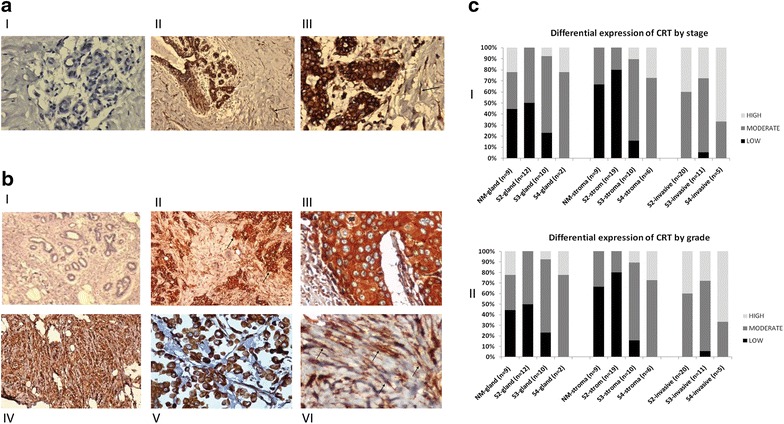
Immunohistochemistry results and the comparison of the expression levels of calreticulin in various invasive states of breast cancer. **a** Immunohistochemical staining of calrecticulin expression in non-malignant lesions of human breast. (*I*) Negative control. (*II*) Positive staining in the glandular (*white arrow*) and stromal compartments (*black arrow*), ×10 magnification. (*III*) Positive staining in glandular (*white arrow*) and stromal compartments (*black arrow*), ×40 magnification. **b** Immunohistochemical staining of calreticulin expression in malignant lesions of human breast. (*I*) Negative control. (*II*) Positive staining in malignant glandular epithelium (*black arrows*) and stromal parts (*white arrows*) of the tumor, ×10 magnification. (*III*) Positive staining in glandular parts of breast tumor. (*IV*) Breast tumor with invasive pattern, ×10 magnification. (*V*) Breast tumor with invasive pattern, ×40 magnification. (*VI*) Calreticulin expression in stromal cells with spindle shapes (*arrows*). **c** (*I*) Differential expression of calreticulin in different stages of breast cancer. Calreticulin expression is presented based on the localization in three different tissue compartments: glandular structures, stroma and invasive areas. The expression of calreticulin is stratified according to the stage of the tumor (2, 3, 4) and the intensity of expression (low, moderate, high). (*II*) Differential expression of calreticulin in different grades of breast cancer. Calreticulin expression is presented based on the localization in three different tissue compartments: glandular structures, stroma and invasive areas. The expression of calreticulin is stratified according to the grade of the tumor (1, 2, 3) and the intensity of expression (low, moderate, high)

To confirm our protein profiling results and characterize the histological pattern of calreticulin expression, we applied IHC on a range of breast cancer specimens with varying degrees of invasive potential. Our results confirmed that in non-malignant tissues, calreticulin expression was more prominent in the glandular compartment as compared to the stroma (Fig. 2a). The expression was mostly cytoplasmic while low intense staining of the nucleus and cell membrane was also detectable. In malignant tissues, calreticulin was expressed in both the epithelial and stromal compartments (Fig. 2b). The expression was high in almost all malignant epithelial cells while inside the tumor stroma, high expression of calreticulin could be observed in spindle shaped cells of the stroma. The pattern of expression revealed that the higher the invasive state of the tissue, the more calreticulin was expressed. To quantify the differences in calreticulin expression among various malignant states, IHC results were scored and the different categories (non-malignant/malignant/stage/grade) were compared (Fig. 2c). One-way ANOVA followed by post hoc test (Duncan option) demonstrated a significantly higher expression of calreticulin in the stromal compartments of malignant tissues as compared to non-malignant tissues; as F(3, 41) = 4.985 and p = 0.005 (Table 1).

**Table 1:** Statistical analysis of immunohistochemistry results for calreticulin expression (ANOVA)

Stage	Sum of squares	df	Mean square	F	Sig.
Glandular tissue					
Between groups	10.854	3	3.618	0.777	0.516
Within groups	139.617	30	4.654		
Total	150.471	33			
Stroma					
Between groups	60.128	3	20.043	4.985	0.005
Within groups	164.85	41	4.021		
Total	224.978	44			
Invasive carcinoma					
Between groups	28.301	2	14.15	3.213	0.053
Within groups	145.338	33	4.404		
Total	173.639	35			

One-way ANOVA showed significantly higher calreticulin expression in stromal parts of malignant tissues as compared to non-malignant samples (p < 0.05)

**Fig. 3:**
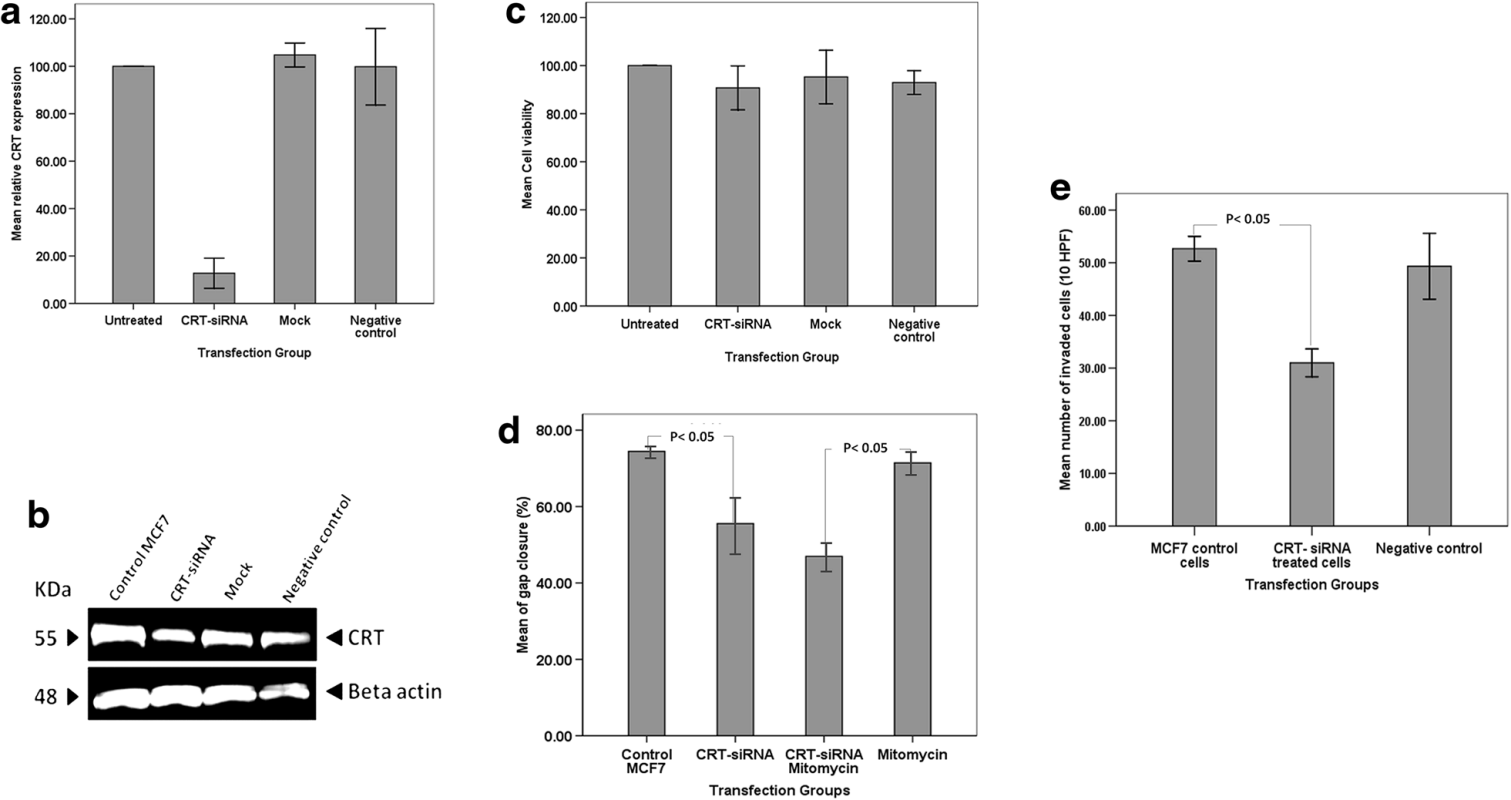
The knockdown of *CRT* expression results in the inhibition of the migratory and invasive potential of MCF7 breast cancer cells. **a** Relative *CRT* expression analysis shows that more than 87 % decrease in expression was achieved in *CRT*-knockdown cells. **b** Western blot results confirmed the down regulation of calreticulin expression in *CRT*-knockdown MCF7 cells as compared to control groups. Beta actin was used as the house-keeping control. **c** MTT assay showed 90.7 % viability in *CRT*-siRNA transfected MCF7 cells as compared to the untreated group. **d** Migration assay results revealed a significant association between *CRT* expression and the migratory potential of MCF7 cells. The effect on migration was not proliferation-dependent. **e** Statistical analysis of the invasion assay results showed a significant decrease in the number of invading cells following *CRT*-knockdown of MCF7 cells

Since IHC confirmed the differential expression of calreticulin in breast cancer tissues, we developed an in vitro *CRT*-knockdown model of breast cancer using siRNA to examine the effects of *CRT* expression modulation on the invasive potential of MCF7 breast cancer cells. The level of *CRT* expression decreased by 87 % following siRNA transfection (Additional file 2: Table S2; Fig. 3a), which confirmed the successful knockdown of *CRT*. This was further confirmed by western blot analysis (Fig. 3b). In addition, MTT assay was used to evaluate the presence of potential cytotoxic effects of the siRNA and transfection reagent. The mean viability of the *CRT*-knockdown cells remained at about 90 % as compared to untreated cells (Fig. 3c).

**Fig. 4:**
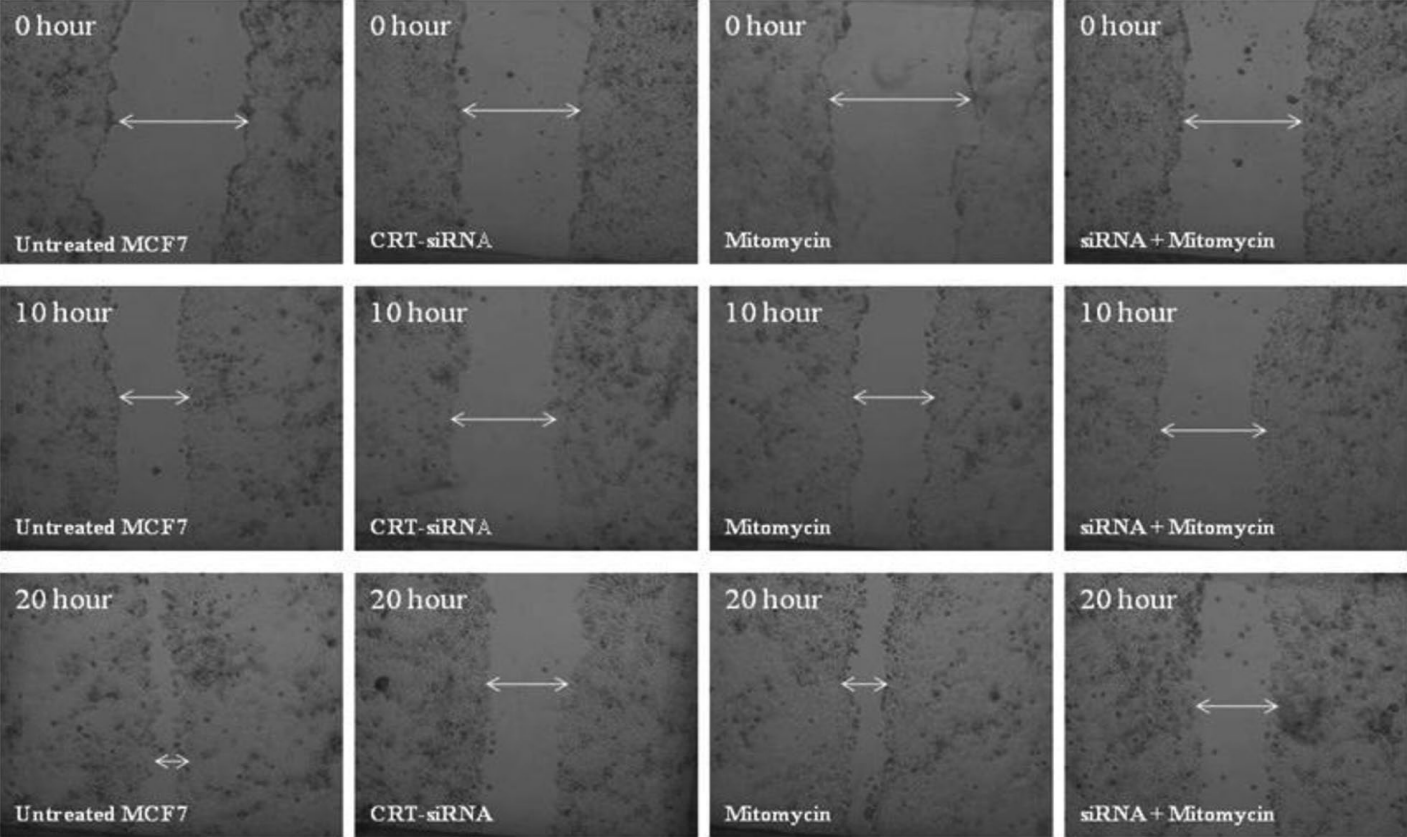
Migration assay: comparing the speed in gap closure in a monolayer of cells. The images were captured at 0, 10 and 20 h post scratching. The *CRT*-siRNA knockdown cells displayed an obvious decrease in gap closure rate as compared to the control group in the absence and presence of mitomycin-C

The invasive characteristics of *CRT*-knockdown cells were then investigated using phenotypic assays. The migration assay (Additional file 3: Table S3; Figs. 3d, 4) revealed a significant decrease in the migratory potential of *CRT*-knockdown cells as compared to the control group (p < 0.05). The treatment with mitomycin-C did not alter this effect, suggesting that the inhibitory effect of calreticulin on migration is not proliferation-dependent (p < 0.05).

**Fig. 5:**
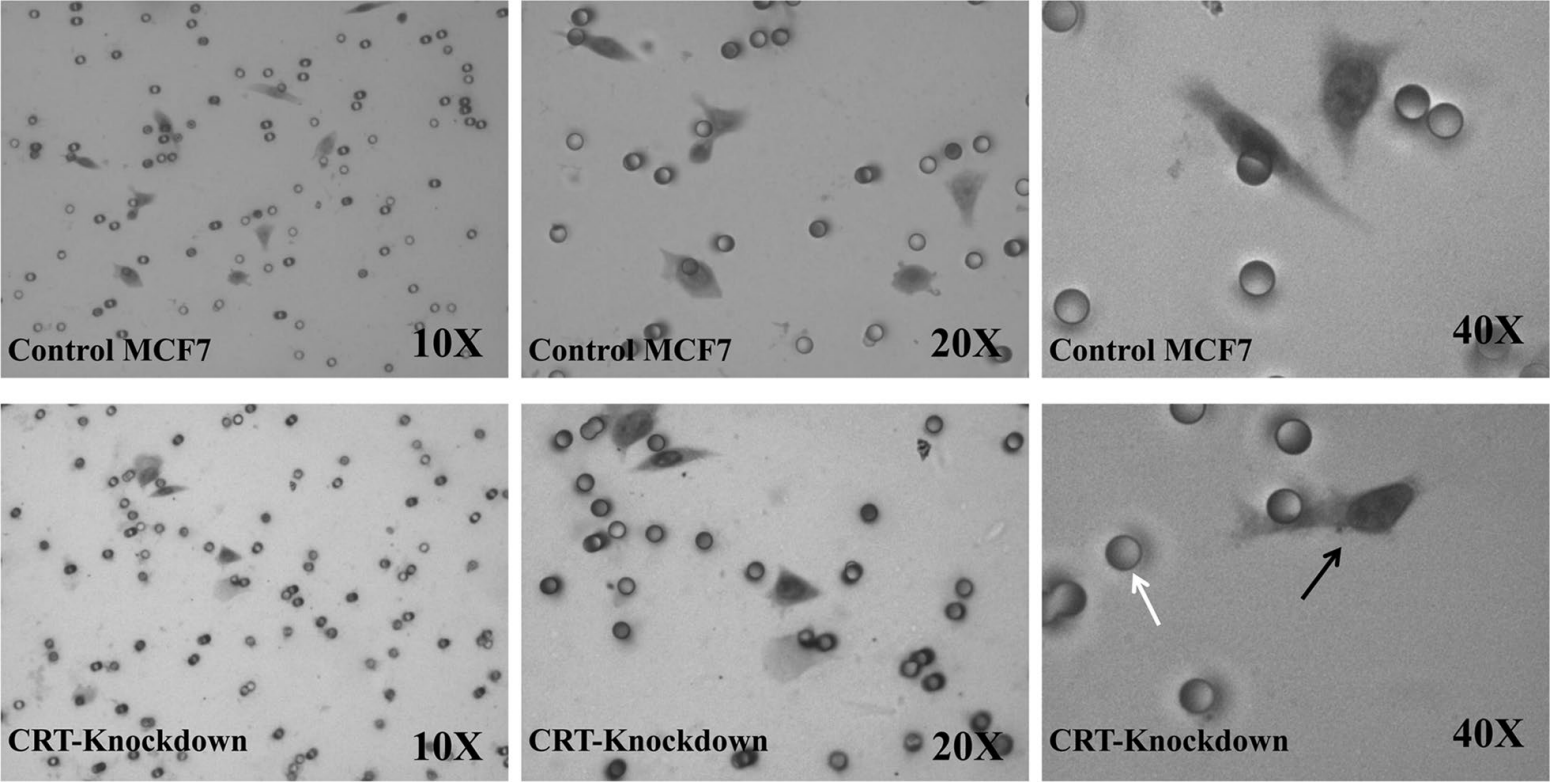
Matrigel invasion assay: micrograph of transwell invaded cells. Control and *CRT*-knockdown MCF7 cells (*black arrow*) are shown to have invaded through the pores (*white arrow*)

The mean number of invaded *CRT*-knockdown cells in a matrigel invasion assay (Additional file 4: Table S4; Fig. 5) was compared against the controls (Fig. 3e). There was a significant decrease (p < 0.05) in the number of invaded *CRT*-knockdown cells as compared to the control cells.

**Fig. 6:**
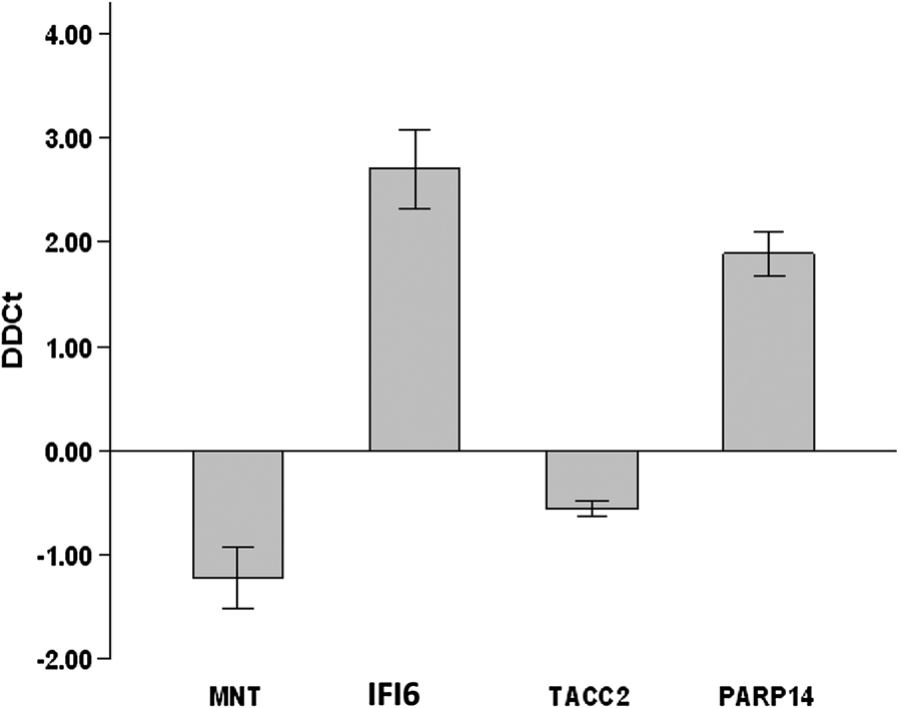
Validation of microarray results by qPCR. The relative expression of selected genes are shown. *IFI6* and *PARP14* were upregulated in *CRT*-knockdown MCF7 cells while *MNT* and *TACC2* were downregulated

We then tried to identify possible *CRT*-associated signaling pathways using gene expression microarrays. A total of 1579 genes were significantly upregulated while 992 genes were downregulated in the *CRT*-knockdown cells. The 10 most up/downregulated genes are summarized in Table 2. The validity of the microarray data was confirmed using qPCR (Fig. 6).

**Table 2:** The 10 most up/downregulated genes upon knockdown of *CRT *expression

Gene name	Systematic name	Log FC	p value
*IFIT1*	NM_001548	2.9	0.00065
*IFIT2*	NM_001547	2.5	2.10E−05
*IFI6*	NM_022873	2.3	0.00016
*IFIT3*	NM_001549	2.3	0.00033
*IFI27*	NM_005532	2.2	0.0034
*DDX58*	NM_014314	2	7.20E−06
*DDX60*	NM_017631	2	5.00E−05
*IFI27*	NM_005532	2	0.0045
*OASL*	NM_003733	1.9	2.10E−05
*SAMD9*	NM_017654	1.9	7.30E−05
***CALR***	**NM_004343**	**−2.7**	**5.20E−15**
*MNT*	AF318360	−2.1	2.30E−13
*CA306742*	CA306742	−1.5	9.30E−12
*FAM35A*	NM_019054	−0.67	4.90E−06
*PCNXL3*	NM_032223	−0.58	2.00E−07
*C13orf37*	NM_001071775	−0.57	2.70E−06
*TACC2*	NM_206862	−0.56	0.00025
*ENST00000301038*	ENST00000301038	−0.5	5.40E−07
*CPOX*	NM_000097	−0.46	0.00037
*TAOK3*	NM_016281	−0.46	0.0023

Genes are listed based on their names, systematic names, fold changes in their expression (log FC) and the p values. CRT as the most down-regulated gene is shown in bold

To understand the biological significance of the global changes in the patterns of gene expression, related gene ontologies were analyzed. GeneCards® which is one of the options of GeneDecks V3 software was used for annotation classification. DAVID (Database for Annotation, Visualization and Integrated Discovery) was also used to reveal functional relationships of the proposed subsets of genes and related ontologies. Some of the most important and related ontologies for up/down-regulated genes are summarized in Additional file 5: Table S5. The genes in each category have been previously reported in the literature as responsible for or involved in the specific ontologies.

Among the significantly upregulated genes, *MIA* (melanoma inhibitory activity), *KLK13* (kallikrein-related peptidase 13), *HEPACAM* (hepatic and glial cell adhesion molecule), *MADD* (MAP-kinase activating death domain), *TP53I11* (tumor protein p53 inducible protein 11), based on the importance of their cellular localization have been reported as being associated with a more invasive cancer phenotype. In addition, *LGALS3BP* (lectin, galactoside-binding, soluble, 3 binding protein), *MAP3K5* (mitogen-activated protein kinase kinase kinase 5), *TNFSF10* (tumor necrosis factor (ligand) superfamily, member 10) which are mainly involved in various cellular processes are potentially associated with a higher invasive potential. Finally, *BTG2* (BTG family, member 2), *RARRES3* (retinoic acid receptor responder 3), *SERPINA3* (serpin peptidase inhibitor, clade A (alpha-1 antiproteinase, antitrypsin), member 3), *SMPD3* (sphingomyelin phosphodiesterase 3, neutral), *XAF1* (XIAP associated factor 1), *SERPINB5* (serpin peptidase inhibitor, clade B (ovalbumin), member 5) and *TP53TG5* are important cancer-related genes that were also significantly up-regulated in this study.

Among the significant downregulated genes, *CCT5* (chaperonin containing TCP1, subunit 5), *DBF4, HDAC4* (histone deacetylase 4), *HDAC4* and *TAF3* with important cellular localizations were shown to be correlated with higher invasive potential. In addition, *CALB2* (calbindin 2) or *calretinin*, *MAPRE2* (microtubule-associated protein, RP/EB family, member 2), *RNASE1* (ribonuclease, RNase A family, 1), ESRRG or estrogen related receptor gamma, MAPK8 (mitogen-activated protein kinase 8) and *PCMT1* (protein-L-isoaspartate (D-aspartate) O-methyltransferase) which are involved in various cellular processes have been reported to be associated with a more invasive cancer phenotype. Finally, *C11orf17* (A kinase interacting protein 1), *MAP2K6* (mitogen-activated protein kinase kinase 6: *MKK6*), *PARD6A* (par-6 partitioning defective 6 homolog alpha), *MINA* (MYC induced nuclear antigen), *NUDT6* (fibroblast growth factor 2: *FGF*-*2*), *PSMD10* (proteasome (prosome, macropain) 26S subunit, non-ATPase, 10: *p28*), *PSMD10*, *FABP3* (fatty acid binding protein 3), *AMPK* (AMP-activated protein kinase) and *PRND* (prion protein 2) have been suggested to potentially contribute towards the migratory and morphological behavioral changes of tumor cells.

We also probed for any significant changes in the expression of other genes related to calreticulin chaperoning or calcium homeostasis. Our results showed significant overexpression of *ERp57*, *GRP78* and *GRP94* in *CRT*-knockdown breast cancer cells. These proteins are actively involved in protein chaperoning inside the ER. In addition, *ERO1A* which codes for an oxidizing enzyme involved in the regulation of the redox state of various ER proteins showed significant upregulation in *CRT*-knockdown genes. Finally, *ITPR3* which encodes the receptor for inositol 1,4,5-trisphosphate was downregulated in *CRT*-knockdown cells. ITPR3 contains a calcium channel and is involved in the release of intracellular calcium.

## Discussion

Our results demonstrate that the level of calreticulin expression in breast cancer is directly correlated with the invasiveness of the malignant lesions; represented by tumor stage/grade. This is in concordance with previous studies in which calreticulin overexpression was described in breast tumor epithelial cells, in which a correlation between calreticulin overexpression and the development of post-operative distant metastasis were reported [23, 24]. Calreticulin overexpression have also been reported in other malignant lesions such as esophageal squamous cell carcinoma [18], gastric cancer [19], pancreatic tumors [17], and even in melanoma cells [21].

The present study also reveals a significant upregulation of calreticulin in the stromal compartment of malignant tissues as compared to non-malignant tissues. To our knowledge, this is the first report that highlights the distinct calreticulin expression in the stroma of breast cancer. It is known that the growth and progression of malignant lesions entail the involvement of tumor stroma [25]. The stroma within the cancer microenvironment is usually subjected to different changes such as fibroblast activation, remodeling of extracellular matrix and finally angiogenesis [26]; where they are thought to act as a machinery for transforming the stroma into a protective microenvironment for metastasis [27]. Among the multiple inter players of the stroma/malignant cellular reaction, cancer-associated fibroblasts are drawing more attention as the receivers and creators of pro-tumorigenic signals [28]. In the present study, the characterization of calreticulin expression in breast cancer tissues shows the potential significance of its expression in the stroma.

The in vitro part of our study demonstrated that the migratory and invasive potential of *CRT*-knockdown MCF7 cells declined significantly as compared to the control groups. At the same time, MTT assay confirmed that these changes were not due to the alterations in the proliferative activity of the cells. These findings are in agreement with recent studies that indicate a role for calreticulin in conferring a pro-invasive and metastatic phenotype in a range of cancers, including breast adenocarcinoma [24, 29], gastric cancer [19], and esophageal squamous cell carcinoma [18].

Although the molecular mechanisms for cancer invasion and metastasis are not fully understood [30], different classes of proteins are involved or altered throughout this process. These proteins include cell adhesion molecules (CAMs) such as calcium dependent cadherin family, integrins and matrix proteases [31]. As calreticulin is also mainly involved in calcium homeostasis and protein chaperoning [5], several mechanisms can be proposed for its role in cancer initiation and progression. Calreticulin is able to prevent the anti-metastatic effects of estrogen receptor α (ERα) in breast cancer [29], induce cell migration and wound healing [32], as well as affect apoptosis through the regulation of calcium stores of the cell [11, 33, 34]. In addition, calreticulin effects on gene expression and cell adhesion are modulated indirectly through Ca^2+^ which makes it a central connector molecule inside a network of signaling pathways that are located in the endoplasmic reticulum [35]. On the cell surface, calreticulin regulates focal adhesion disassembly through the interaction with TSP/hep I complex suggesting it as an important component of the TSP signaling pathway that modulates cytoskeleton structure and supports the role in cell adhesion [36].

To propose a model of *CRT* gene interactions and related molecular pathways involved in conferring a more invasive breast cancer phenotype, further bioinformatic analysis was performed. Functional annotation of differentially expressed genes in *CRT*-knockdown cells revealed several dysregulated biological processes which can be suggested to be involved in calreticulin-associated pro-invasive effects. The most important dysregulated processes amongst the top gene ontologies were extracellular region, calcium homeostasis, DNA binding, regulation of transcription, estrogen metabolism and pathways, tumor progression, metastasis and cancer related processes. As expected, among the genes that were significantly up or downregulated, there were components of important signaling pathways such p53 and MAPK (Table 3) in which their possible interactions with calreticulin may contribute towards a more invasive breast cancer phenotype.

**Table 3:** The fold-change in gene expression of p53 and MAPK related genes from the microarray-based whole genome expression analysis of *CRT*-knockdown of MCF7 cells

Pathway	Gene name	Systematic name	Log FC	p value
MAPK	*MAP2K6*	NM_002758	−0.37	0.00091
	*MAPK8*	ENST00000374189	−0.16	0.031
	*MAP2K1 (MKK1)*	NM_002755	−0.12	0.31
	*MAP2K4 (MKK4)*	NM_003010	−0.11	0.18
	*MAPK1 (ERK2)*	NM_138957	−0.01	0.96
	*MAPK14 (p38)*	NM_001315	−0.08	0.39
p53	*SERPINB5*	NM_002639	0.18	0.032
	*TP53I11*	NM_001076787	0.25	0.036
	*TP53TG5*	NM_014477	0.21	0.011

**Fig. 7:**
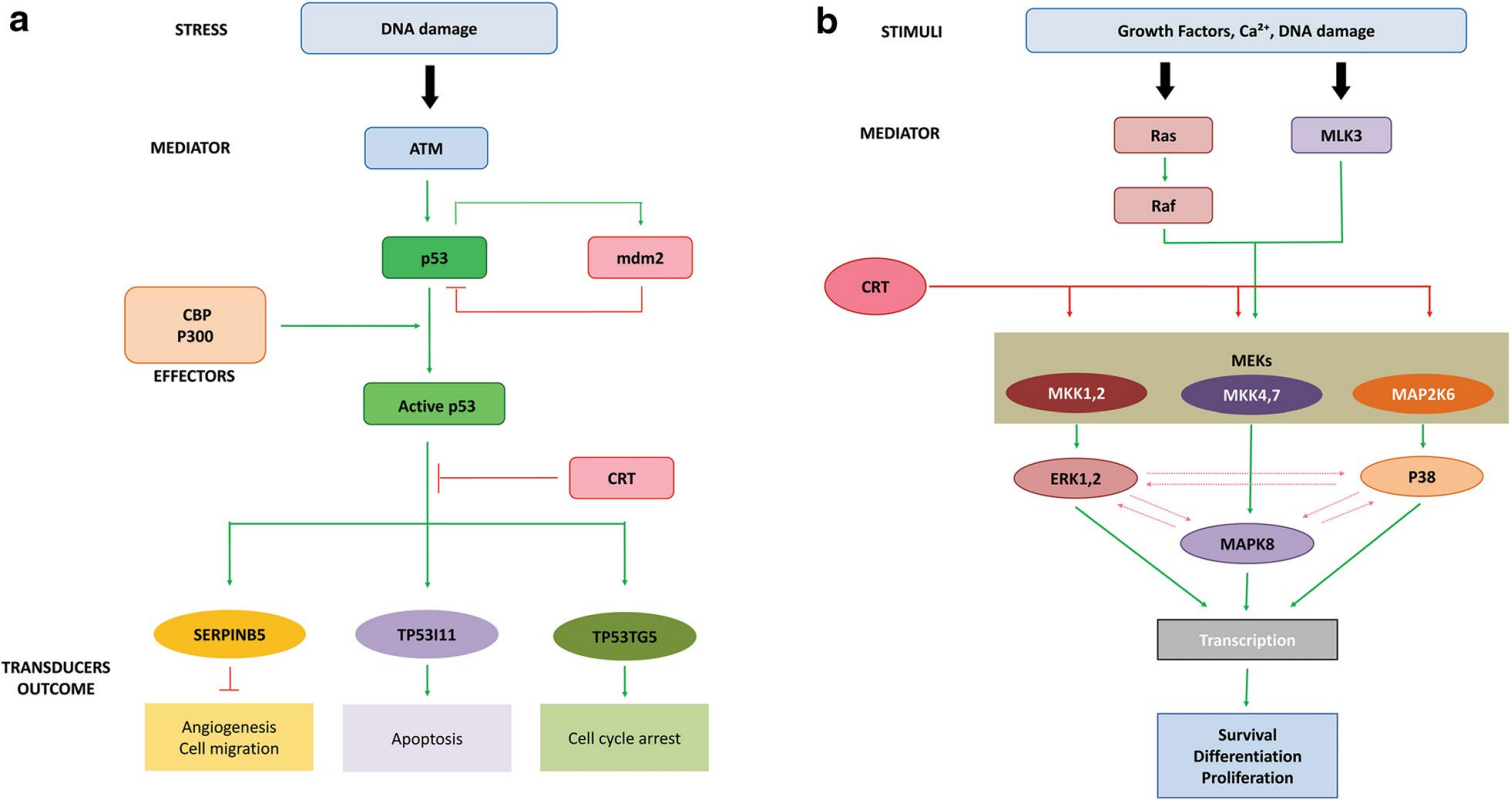
*CRT*-knockdown correlates with the modulation of p53 and MAPK pathways. **a** Possible role of calreticulin in the p53 pathway. Activated p53 suppresses the processes of tumor formation and progression through the activation of SERPINB5, TP53I11 and TP53TG5. Calreticulin possibly blocks p53 stimulatory effects of these processes. **b** Possible contribution of calreticulin in the MAPK pathway. MEKs are activated following the attachment of growth factors and other ligands to the surface receptors. This leads to the activation of sequential phosporylation processes, enhancement of transcriptional activities and finally regulation of gene expression. MAP2K6 and MAPK8 are two significantly modulated MAPKs following *CRT* knockdown (p < 0.05) while MKK1 and MKK4 have also shown some changes in their expression levels. Calreticulin possibly has a stimulatory effect on the production of MKKs

In the p53 pathway, active p53 functions as a transcription factor that upon sensing DNA damage or stress, will trigger a network of genes and their products such as TP53I11, TP53TG5 and SERPINB5 (maspin) that ultimately leads to cell cycle arrest, cellular senescence or apoptosis [37]. Our gene expression study confirmed that all these three p53 targets were significantly modulated following *CRT *knockdown in MCF7 cells (Fig. 7a). *TP53I11* is a tumor suppressor gene that is involved in apoptosis [38]. *TP53TG5* is a novel p53 target gene with cell cycle localization and growth suppressive effects in glioblastoma cells [39]. Finally, *SERPINB5* (maspin) is a powerful tumor suppressor gene that is regulated by p53 at the transcriptional level and inhibits cell motility, invasion, angiogenesis and metastasis [40]. SERPINB5 is downregulated in breast cancer [41], and has the ability to modify the motility of breast cancer cells in vitro by inhibiting Rac1/Cdc42 activity [42]. In addition, SERPINB5 can potently inhibit the development of brain metastasis of breast cancer [43].

Our novel finding; significant upregulation (p < 0.05) of the three p53 target genes following *CRT*-knockdown correlates with the inhibition of the migratory and invasive potential of breast cancer cells. Therefore, we postulate that calreticulin is possibly able to modify the inductive effects of activated p53 on its target genes. The mechanism behind calreticulin-blocking effect on p53 target genes is not clear. However, it can be related to the changes in Ca^2+^ homeostasis as p53 has been reported to interact with other Ca^2+^ binding molecules such as S100A2 [44].

The activation of MAPK signaling through the extracellular signal related kinase pathway (ERK1, 2) and or stress activated kinase pathway (JNK/p38 MAPK) will lead to sequential phosphorylation events (Fig. 7b), activation of transcription factors and finally gene expression regulation [45]. Erk1 and 2 are the most relevant MAPK components for breast cancer development [46], while the level of activated p38 expression is reported to be associated with the presence of lymph node metastasis in breast cancer [47].

Our results suggest that *CRT* down-regulation in MCF7 cells will result in significant downregulation (p < 0.05) of the expression levels of some of the components of MAPK pathway including *MAP2K6* and *MAPK8*. In addition, some of the other MAPK components such as *MEK1* and *MEK4* also showed reduction in their level of expression. These findings correspond to the significant decrease in the migratory and invasive potential of the cells. Therefore, calreticulin can be proposed to act as a potential inducer of the MAPK pathway. This can be supported by the fact that MAPK pathway activity is also modified by other Ca^2+^ homeostasis proteins such as Ras-GRF1, which is a Ca^2+^/calmodulin-dependent Ras-guanine-nucleotide-releasing factor [45], SERCA2 [48], and CaMKII (calcium/calmodulin-dependent protein kinase II gamma) [49].

## Conclusion

This study shows that calreticulin expression in breast cancer is correlated with the degree of invasiveness; especially when stromal expression is considered. Furthermore, calreticulin confers a more invasive breast cancer phenotype. We conclude that calreticulin is able to promote breast cancer migration and invasion through the transcriptional modulation of important genes and associated pathways such as MAPK and p53 signaling. These downstream genes that have an active contribution towards the malignant transformation of cancer cells are possibly the actual targets of calreticulin-associated pro-invasive effects and will need to be further evaluated in future studies.

## Methods

### Human breast tissue samples and breast cancer cell line

Fresh frozen breast cancer tissues for this study were obtained from Hospital Universiti Kebangsaan Malaysia. Ethics approval and patient informed consent including consent to participate in the study and consent to publish was obtained in written in accordance to the Universiti Kebangsaan Malaysia Research and Medical Ethics Committee (Approval No. FF-166-2004). Fresh frozen samples of infiltrating ductal carcinoma and adjacent normal tissues were collected from 12 patients who underwent breast surgery. The samples were snap frozen and stored at −80 °C until protein analysis was performed. Formalin-fixed paraffin-embedded (FFPE) samples were obtained from the Pathology Department of Milad Hospital, Tehran, Iran, courtesy of Dr. Saadat Molanaii. These included 10 non-malignant and 38 malignant samples. Non-malignant samples were mainly from fibrocystic changes of the breast while all of the malignant tissues were diagnosed as ductal carcinoma. Malignant samples were stratified based on the grade as well as the stage of the tumors. MCF7 cell line was purchased from the American Type Culture Collection (ATCC) and used for the in vitro part of this study,

### Two-Dimensional Gel Electrophoresis

For the first dimension, 17 cm pH 4–7 L IPG strips (Bio-Rad, Hercules, CA, USA) were used. The samples were passively rehydrated overnight using a rehydration buffer containing 7 M urea (Bio-Rad), 2 M thiourea (GE healthcare, Uppsala, Sweden), 2 % CHAPS (GE Healthcare), 1 % IPG buffer (pH 4–7) (GE healthcare), 0.2 % DTT (Bio-Rad) and bromophenol blue (Bio-Rad). The rehydrated IPG strips were focused using PROTEAN IEF cell (Bio-Rad). The current was applied in 3 steps; 250 V was applied in linear ramp for 20 min, then 10,000 V for 2.5 h in linear ramp and finally, the current increased rapidly to 40,000 V/hr when the isoelectric focusing was achieved. The strips were stored at −80 °C until the second dimension gel electrophoresis was performed. Strips were then equilibrated in two steps for 15 min each using an equilibration buffer consisting of 0.1 M Tris HCl (Amresco, Solon, OH, USA) pH 8.8, 6 M urea (Bio-Rad) 30 % Glycerol (GE Healthcare), 4 % SDS (GE Healthcare), 0.002 % Bromophenol Blue (Bio-Rad). In the first step of equilibration, 26 mM DDT (Bio-Rad) was added to the buffer, while 0.38 M of Iodoacetamide (Bio-Rad) was added to the buffer in the next step. The second dimension was then performed on 10 % SDS–polyacrylamide gels.

### Gel staining and analysis

The gels were stained with silver staining and scanned using the GS-800 Calibrated Densitometer (Bio-Rad). The PDQuest software 7.3 (Bio-Rad) was used for differential analysis of the gels. The spots with at least a twofold increase were picked manually and stored at 4 °C until in-gel digestion was carried out.

### Protein identification by MALDI-TOF MS

The excised spots were destained by first washing with a digestion buffer (25 mM ammonium bicarbonate) and then with 50 % acetonitrile digestion buffer. These steps were repeated three times. Digestion was performed with sequencing grade trypsin (Promega, Madison, WI, USA) at 37 °C overnight. The resulting tryptic peptides were extracted from the gels, then desalted and concentrated with C18 ziptip (Millipore, Billerica, MA, USA) before spotting for matrix-assisted laser desorption ionization-time-of-flight (MALDI-TOF) analysis.

### Immunohistochemistry

Immunohistochemistry was performed to evaluate histological expression of calreticulin using a mouse monoclonal [FMC 75] antibody (Abcam, Cambridge, UK) through the following protocol: 0.4 µ tissue sections were mounted on polysine-coated slides (Menzel, Berlin, Germany). After dewaxation and dehydration, heat induced antigen retrieval was done using Tris–EDTA buffer (pH: 9.0) for 10 min in sub-boiling conditions. This was followed by internal peroxidase inhibition with 3 % H2O2 for 10 min, blocking with 5 % BSA for 2 h, and then treatment with a primary antibody (anti-calreticulin) for 45 min at a concentration of 0.15 µgr/mL. For detection, Real™ EnVision™ Detection System (Dako, Glostrup, Denmark) was used for 30 min followed by chromogen staining using diaminobenzidine (DAB) for 10 min and finally counterstained by hematoxylin. All the procedures were carried out at room temperature.

### Scoring the IHC results

An independent committee blinded to pathologic details of the specimens was assigned to score the IHC results. Three different histological areas of each malignant tissue including glandular, invasive and stromal parts were examined. These scores were calculated based on the intensity of the brown staining (1–3 plus) as well as the proportion of stained cells in glandular epithelium (<50 % = 0, 70–50 % = 1, 90–70 % = 2 and >90 % = 3) as well as stroma (<10 % = 1, 10–50 % = 2 and >50 % = 3).

### RNA isolation & cDNA synthesis

Total RNA extraction was performed using RNeasy mini kit (Qiagen, Valencia, CA, USA) following the manufacturer's protocol. The concentration and quality of RNA were examined by measuring the absorbance ratios at 260 nm and 280 nm using the NanoDrop 2000 spectrophotometer (Thermoscientific, West Palm Beach, FL, USA) and running the samples on 1 % agarose gel, respectively. Finally, the RNA samples were reverse transcribed into cDNA using the Revert Aid™ H minus First Strand cDNA Synthesis kit (Thermoscientific) according to the manufacturer’s instructions.

### Real-time PCR

The expression level for *CRT* as the gene of interest and *beta actin* as the housekeeping (reference) gene were examined using the SensiMix*Plus* SYBR kit (Quantace, London, UK) and then quantified based on the standard curve method. The primers were designed in a manner to amplify only the cDNAs compatible to the mRNA sequence but not the genomic sequence. The sequences of the primers for *CRT* are as follows: GGCGGCACCACCATGTACCCT (forward) and AGGGGCCGGACTCGTCATACT (reverse) while that for *beta-actin* are CTTCAAGGAGCAGTTTCTGG (forward) and GCTTGTCTGCAAACCTTTATC (reverse). The protocol for PCR was as follows: initial denaturation at 95 °C for 10 min, denaturation at 95 °C for 30 s, annealing at 52 °C (59 °C for beta actin) for 30 s, elongation at 72 °C for 45 s, and final elongation at 72 °C for 10 min. The reaction was run for 35 cycles.

### Protein isolation

MCF7 cells were cultured until they reached 70–90 % confluence and were then lysed with a solution of 40 mM Tris (GE Healthcare, WI, USA), 7 M urea (Invitrogen, Carlsbad, CA, USA), 2 M thiourea (Invitrogen), 4 % CHAPS (Invitrogen), 65 mM Dithiothreitol (Invitrogen), 1 mM EDTA (Amresco) and 10 μl of Halt Protease Inhibitor Cocktail (Thermoscientific).

### Western blot

The isolated proteins were run on 12.5 % polyacrylamide resolving gel and then transferred to a PVDF membrane and treated with the primary antibody at 4 °C overnight either using 1:200 dilution of mouse monoclonal antibody against human calreticulin 1G6A7 (Santa Cruz Biotechnology, Santa Cruz, CA, USA) or 1:10,000 dilution of mouse monoclonal [Ac-15] antibody against human beta actin (Genetex, Irvine, CA, USA). This was followed by an incubation in 1:2500 dilution of the secondary antibody (ImmunoPure Goat Anti-Mouse IgG, (H + L), Peroxidase Conjugated, Thermoscientific). The detection was carried out by a chemiluminescence method using SuperSignal West Pico Chemiluminescent Substrate (Thermoscientific).

### siRNA gene knockdown

For *CRT* knockdown, ON-TARGET*plus* SMARTpool (Thermoscientific) and *DharmaFECT* 1 as the transfection reagent were used. Three controls were included in the experiment including untreated, negative and mock controls. For negative control (NC), ON-TARGET*plus* SMARTpool was replaced by non-targeting pool of siRNA. In mock control (M), ON-TARGET*plus* SMARTpool was replaced by equal amounts of antibiotic- and serum-free media. Finally, ON-TARGET*plus* SMARTpool and *DharmaFECT* 1 were replaced by antibiotic- and serum-free media in the untreated control (UT). For mRNA analysis, cells were harvested 36 h post-transfection while protein extraction was carried out at 72 h post-transfection.

### MTT assay

MCF7 cells were cultured on 96-well plates in triplicates containing *CRT*-siRNA transfected or related control groups. At 24 h post-transfection, 20 μl of 5 mg/ml MTT solution was added to each well and incubated for 3.5 h at 37 °C and 5 % CO_2_. The content of each well was then replaced with 100 μl of DMSO (dimethyl sulfoxide). The plate was covered with an aluminium foil and agitated in an orbital shaker at room temperature for 15 min. Finally, the absorbance was read at 590 nm with a reference filter at 620 nm using an ELISA reader.

### Migration assay

Four triplicates of MCF7 cells were grown on 12-well plates and incubated at 37 °C and 5 % CO_2_ until the cultures reached 70 % confluency. Two sets of cells were transfected with *CRT*-siRNA and the cultures were kept at 37 °C and 5 % CO_2_ for another 72 h. Then, 10 μg/ml mitomycin C (Merck, Whitehouse Station, NJ, USA) was added to one set of each triplicate (both siRNA treated and untreated). After 2 h, a longitudinal scratch was made on the central line of each well alongside the pre-applied marks at the bottom of each plate. Using a microscope with an attached camera (Leica, Wetzlar, Germany), images were captured from the specified marked loci of the gaps that were produced by the scratch on the monolayer of cells. Image capturing was repeated at certain time points including 0, 3, 6, 10, 15, 20, 24, 28, 32 and 36 h post-scratching. Finally, the diameter of the gap was measured in three different loci of each replicate at the specified marked areas using the Nis-Elements imaging software version 3.00 SP7. The average readings were calculated and the proportion of differences in the gap diameter at 0 and 20 h were used to compare the migratory potential of the different groups.

### Invasion assay

At 48 h post-transfection, the media was replaced by serum-free DMEM overnight to simulate starving conditions. The following day, cells were harvested and diluted at a concentration of 1 × 10^5^ cells/mL. The lower compartment of BD companion plates (BD, Franklin Lakes, NJ, USA) were filled with 750 μl of DMEM including 10 % FBS. Then, matrigel-coated inserts were placed inside the wells and 500 μl of cell suspension (5 × 10^4^ cells) was added into each well and incubated at 37 °C and 5 % CO_2_ for 24 h. The inserts were then incubated in 100 % methanol for 10 min, washed in PBS twice and stained in hematoxylin for 2–5 min. Finally, the filter membranes were cut and mounted on slides using DPX (Merck). The total number of invaded cells in 10 random HPF was counted at 10× magnification of a light microscope.

### Genome wide expression analysis

A triplicate hybridization of *CRT*-siRNA transfected MCF7 cells versus mock control was prepared and total RNA was extracted 36 h post-transfection. Only RNA samples with high quality (A260/A280 and A260/230 were set at ≥1.8 and ≥1.5, respectively) were picked for further evaluation. Agilent Whole Human Genome Microarray 4 × 44 K G4112F (Agilent, Palo Alto, CA, USA) which, consisted of 43,376 oligonucleotides was used for expression profiling. The preparation of expression arrays, hybridization process and scan readings were carried out by Oxford Gene Technology (Oxfordshire, UK). The microarray data was deposited in NCBI’s Gene Expression Omnibus [50] and is accessible through GEO Series accession number GSE44371.

### Statistical analysis

For the IHC results, one-way ANOVA followed by post hoc test (Duncan option) was used. In other experiments, independent Student’s t test was used to determine any statistical significance of the results.

## Additional files

10.1186/s12935-016-0329-y Protein identification of the excised spots.
10.1186/s12935-016-0329-y Relative quantification of *CRT *expression in MCF7 cells.
10.1186/s12935-016-0329-y Migration assay results.
10.1186/s12935-016-0329-y Invasion assay results.
10.1186/s12935-016-0329-y The most important gene ontologies related to a subset of significant dysregulated genes.

## Abbreviations

CRT: calreticulin
2D: two dimensional
MALDI-TOF: matrix-assisted laser desorption/ionization time of flight
FFPE: formalin-fixed paraffin-embedded
ER: endoplasmic reticulum
siRNA: small interfering RNA
HPF: high power field
qPCR: quantitative polymerase chain reaction
IDC: intra ductal carcinoma
IHC: immunohistochemistry
nm: nanometer
