# Supplementary material for: Calreticulin mediates an invasive breast cancer phenotype through the transcriptional dysregulation of p53 and MAPK pathways
Source: Cancer Cell Int. 2016 Jul 13;16:56. doi: 10.1186/s12935-016-0329-y (PMC4944499; doi:10.1186/s12935-016-0329-y)
Supplement: Supplementary file 1 — 10.1186/s12935-016-0329-y Protein identification of the excised spots. [file 12935_2016_329_MOESM1_ESM.docx]

**Table S1. Protein identification of the excised spots.**

| **Spot** | **Protein Identified** | **Expression in tumor tissues** | **pI** | **M. Wt (kDa)** | **Coverage (%)** |
| --- | --- | --- | --- | --- | --- |
| 1 | Tumor rejection antigen (gp96) | **↑** | 4. 8 | 92.45 | 24.4 |
| 2 | Calreticulin precursor | **↑** | 4.3 | 48.12 | 30.7 |
| 3 | Chaperonin ( HSP60) | **↑** | 5.7 | 61.04 | 31.4 |
| 4 | T-complex protein 1 (TCP1) | **↑** | 6.0 | 60.33 | 14.7 |
| 5 | Transferrin | **↑** | 7.1 | 77.06 | 15.0 |
| 6 | Protein tyrosine phosphatase | **↓** | 7.0 | 84.46 | 14.9 |
| 7 | Collagen α 1 (VI) precursor | **↓** | 5.3 | 108.51 | 10.0 |
| 8 | Leukocyte-derived arginine aminopeptidase short form | **↓** | 5.8 | 60.92 | 11.1 |
| 9 | Cytokine induced apoptosis inhibitor 1 | **↑** | 5.5 | 39.98 | 20.2 |
| 10 | Actin γ 2 (ACTG2) | **↓** | 5.3 | 41.86 | 29.8 |

The table lists the excised spots from normal and tumor tissues of infiltrating ductal carcinoma, in which 6 spots were found to be over-expressed and 4 spots were down-regulated in tumor tissues.
