# Supplementary material for: Calreticulin mediates an invasive breast cancer phenotype through the transcriptional dysregulation of p53 and MAPK pathways
Source: Cancer Cell Int. 2016 Jul 13;16:56. doi: 10.1186/s12935-016-0329-y (PMC4944499; doi:10.1186/s12935-016-0329-y)
Supplement: Supplementary file 2 — 10.1186/s12935-016-0329-y Relative quantification of CRT expression in MCF7 cells. [file 12935_2016_329_MOESM2_ESM.docx]

**Table S2. Relative quantification of CRT expression in MCF7 cells.**

| **Group** | **CRT**  **(CT)** | **SD** | **CRT Con.**  **(ng/μL)** | **Beta Act.**  **(CT)** | **SD** | **Beta act. Conc.**  **(ng/μL)** | **CRT/Beta actin**  **(Con.)** | **X-folds**  **to calibrator** | **% of CRT**  **expression** |
| --- | --- | --- | --- | --- | --- | --- | --- | --- | --- |
| **treated control** | 11.44 | ±0.04 | 278.46 | 7.14 | ±0.09 | 412.56 | 0.67 | 1.00 | **100.00** |
| **CRT-siRNA treated** | 14.47 | ±0.02 | 34.00 | 7.15 | ±0.12 | 410.89 | 0.08 | 0.12 | **12.26** |
| **Mock control** | 12.39 | ±0.07 | 144.09 | 8.07 | ±0.01 | 198.79 | 0.69 | 1.03 | **102.92** |
| **Negative control** | 12.43 | ±0.03 | 140.13 | 8.06 | ±0.13 | 200.26 | 0.68 | 1.01 | **101.45** |

Table shows the relative expression of CRT gene in CRT-siRNA knockdown cells and control groups. Each group consists of an experimental triplicate. The CRT-siRNA transfected group shows more than 87% knockdown.
