# Supplementary material for: Calreticulin mediates an invasive breast cancer phenotype through the transcriptional dysregulation of p53 and MAPK pathways
Source: Cancer Cell Int. 2016 Jul 13;16:56. doi: 10.1186/s12935-016-0329-y (PMC4944499; doi:10.1186/s12935-016-0329-y)
Supplement: Supplementary file 3 — 10.1186/s12935-016-0329-y Migration assay results. [file 12935_2016_329_MOESM3_ESM.docx]

**Table S3. Migration assay results.**

| **Groups** | **Gap diameter at 0 hr (a)** | **Gap diameter at 20 hr (b)** | **a/b** | **% of closure** | **SD** |
| --- | --- | --- | --- | --- | --- |
| Untreated MCF7 | 500.6 | 127.4 | 4.0 | 74.4 | ±4.2 |
| CRT-siRNA | 556.3 | 248.4 | 2.3 | 55.5 | ±6.4 |
| Mitomycin | 504.6 | 146.4 | 3.7 | 71.4 | ±5.9 |
| CRT-siRNA + Mitomycin | 520.5 | 174.1 | 3.3 | 67.1 | ±6.8 |

Table shows the average percent of gap closure at 20 hours post-scratches.
