# Supplementary material for: Calreticulin mediates an invasive breast cancer phenotype through the transcriptional dysregulation of p53 and MAPK pathways
Source: Cancer Cell Int. 2016 Jul 13;16:56. doi: 10.1186/s12935-016-0329-y (PMC4944499; doi:10.1186/s12935-016-0329-y)
Supplement: Supplementary file 4 — 10.1186/s12935-016-0329-y Invasion assay results. [file 12935_2016_329_MOESM4_ESM.docx]

**Table S4. Invasion assay results.**

| **Groups** | **Mean Cell counts (10 HPF)** | **SD** |
| --- | --- | --- |
| **Control MCF7** | **52.6** | **± 2.2** |
| **CRT-siRNA** | **31** | **± 3.1** |
| **Negative control** | **4.93** | **± 2.5** |

Table shows the mean counts of invaded cells through matrigel filter membrane per 10 high power fields (HPF) microscopy.
