# Supplementary material for: Calreticulin mediates an invasive breast cancer phenotype through the transcriptional dysregulation of p53 and MAPK pathways
Source: Cancer Cell Int. 2016 Jul 13;16:56. doi: 10.1186/s12935-016-0329-y (PMC4944499; doi:10.1186/s12935-016-0329-y)
Supplement: Supplementary file 5 — 10.1186/s12935-016-0329-y The most important gene ontologies related to a subset of significant dysregulated genes. [file 12935_2016_329_MOESM5_ESM.docx]

**Table S5. The most important gene ontologies related to the subset of significant dysregulated genes.**

| **Ontology** | ***Related dysregulated genes (p<0.05)*** | |
| --- | --- | --- |
| Extra Cellular Region | | *ACPL2, ADAM28, ADAMTSL3, APOL2, ARSF, ART5, C2orf69, CEACAM1, CNTNAP3, COL4A4,CPA5, DEAF1, EGFR, EMID1, EPGN, ERAP1, ERBB3, GDF6, IL25, ITIH2, KLK13, LGI4, LOC339240,LYG1, MIA, OMD, OTOA, PCSK6, PGLYRP2, PI15, PROCA1, PROL1, PTGFR, REG3A, REN, SAA3P,SCUBE1, SERPINB5, SPACA3, TAC1, TRYX3, VWF, WFDC11, WNT6, XYLT1* |
| Protein binding | | *ADAR, B2M, BMP7, BST2, BTG2, C1orf38, C3, CAPN1, CEACAM1, CP, CRELD1, DDX58, DSC1, EIF2AK2,ETV4, ETV7, FAM3C, GBP1, HLA-B, HLA-C, HLA-F, HSP90B1, HSPA5, ICAM1, IFI27, IFI6, IFIH1, IFIT3, IL15,IRF1, IRF7, IRF9, ISG15, LAMP3, LAP3, LGALS3BP, LMO2, LTB, MAP3K5, MAPK3, MLL, MLL4, MUC5AC,MX1, OAS1, OAS3, ONECUT2, PDIA3, PML, PNPT1, POLQ, PSMB8, PSMB9, RARRES3, RBBP6, S100A9,SERPINA3, SMG1, SMPD3, SOCS1, SP100, STAT1, TAP1, TMPRSS4, TNFSF10, TRIM25, UBD, UBR4, XAF1,AFF4, AGAP2, AMOTL1, ARG2, ARL4A, ARL6IP6, ATP1B3, BCL2, BMPR1B, C11orf17, CALR, CCR1, CCT5,CDC42, CDC42SE2, CFTR, CMBL, CNGB3, COMMD1, COMMD3, CSTF3, CUL4A, DBF4, DLX2, EIF2C3, EPB41,ERC2, ERGIC1, EXOSC2, FANCF, FEZ2, FLCN, FMNL3, GNA13, GRK5, HDAC4, HIST2H2BE, HSPD1, KHK,KLHL23, KLHL29, KLHL7, KLK8, KRT13, KRT15, KRT35, KRT78, LCK, MAP2K6, MNT, MTMR7, NELL2, NGLY1,NUFIP1, NUMB, OAZ1, PARD6A, PCBD2, PHF17, PM20D2, PRMT2, PSMD10, PSPH, PTPN1, RAN, RNF125,RNF219, RSL1D1, SEC61B, SIAH2, SYN3, SYTL5, TAF11, TAF3, TCEA1, TCTEX1D2, TMEM48, TPM3, TRIM35,TRIM37, TRIM59, TRIM9, TSN, UBE2L3, USHBP1, WDR12, WIPF1, ZBTB2, ZBTB41, ZC3H7B, ZNHIT6* |
| Calcium Homeostasis | | *ABCA1, ADCY5, AHNAK, B2M, BMP7, C3, CAPN1, CEACAM1, CGN, CHD7, CP, CX3CL1, EIF2AK2, ETV7,HSP90B1, HSPA5, ICAM1, IFI35, IL15, IRF1, LAP3, LGALS3BP, MAP3K5, MAPK3, MDFI, MUC5AC, MX1,OBSCN, PDIA3, PDIA4, PLSCR1, PSMB8, S100A9, SERPINA3, SMG1, SMPD3, SOCS1, STAT1, SYT5,TNFSF10, UBD, BBS9, BCL2, CALB2, CALCRL, CALR, CCL15, CCR1, CDC42, CFTR, CGA, CPOX, CYP3A5, DBP, DHFR, EPB41, EPHX2, FLCN, FLG, GLRX, GPR68, GRK5, GTDC1, HDAC4, HSPD1, KCNQ2, KLK8, KRT13, KRT15, LCK, MAP2K6, MAPRE2, MBP, MTMR6, MYH14, NUMB, PSPH, PTPN1, RAN, RNASE1, SIAH2, SLC12A3, SOAT1, SRI, TACR3, TNNI1, TPM3, WIPF1* |
| DNA Binding | | *AKAP8L, ARID3B, ARID4B, CHD7, E2F6, EP400, ETV5, HIC2, HMGA1, HUWE1, KHSRP, MLL3, MLXIP,MYO18A, NR4A1, OVOL1, PML, POU5F1, PRIC285, PRKDC, RARG, RBCK1, RYBP, SALL4, SETX,SRCAP, TBRG1, TNRC18, TRIM33, ZNF100, ZNF429, ZNF493, ZNF66, ZNF680, ZNF768* |
| Regulation of Transcription | | *ARID3B, ARID4B, BRWD1, CAND1, CHD7, CRY2, FRYL, GTF2I, HIC2, JHDM1D, KHSRP, MACC1,MAMLD1, MOV10, MYCBP2, OVOL1, PHF11, PLAGL2, PRIC285, RYBP, SALL4, SETD1B, SRCAP,SUV420H2, TRRAP, ZMYM2, ZNF493, ZNF768* |
| Transcription Factor Activity | | *ARID3A, C5orf41, CBFA2T3, E2F5, E2F6, EGR4, ESR1, ETV5, GLI3, GTF2I, HHEX, HMGA1, MAFF,NFAT5, NFE2L3, NR4A1, PBX4, PLAGL2, POU5F1, RARG, RBCK1, RUNX2, SIX4, SOLH, SPEN,STAT2, ZFHX2, ZNF500* |
| Estrogen Metabolism and Pathways | | *ADH1A, ADIPOQ, CA6, CHRNA4, CRABP1, CRHBP, CTSE, CYP19A1, DRD2, ESRRG, GAL3ST1, GPD1, GRIN2B, HLA-G, HTR4, IL10, LEFTY2, MAP2, MAPK8, NPY2R, NUP43, SLC6A2, SLCO1A2, SNAP25* |
| Progesterone Metabolism and Pathways | | *ADIPOQ, ATM, CA6, CCR3, CHRM2, CHRNA4, CRABP1, CRHBP, CTSE, CYP19A1, DRD2, GPD1, HLA-G, IL10, LEFTY2, MAP2, MAPK8, NPY2R, NUP43, PCMT1, PSMD4, RELN, SERPINB5, SLC6A2, SNAP25, TTF2, TWIST1* |
| Cancer | | *ADAR, B2M, BMP7, BST2, BTG2, C1orf38, C3, CAPN1, CEACAM1, CP, CRELD1, DDX58, DSC1, EIF2AK2, ETV4, ETV7, FAM3C, GBP1, HLA-B, HLA-C, HLA-F, HSP90B1, HSPA5, ICAM1, IFI27, IFI6, IFIH1, IFIT3, IL15, IRF1, IRF7, IRF9, ISG15, LAMP3, LAP3, LGALS3BP, LMO2, LTB, MAP3K5, MAPK3, MLL, MLL4, MUC5AC, MX1, OAS1, OAS3, ONECUT2, PDIA3, PML, PNPT1, POLQ, PSMB8, PSMB9, RARRES3, RBBP6, S100A9,SERPINA3, SMG1, SMPD3, SOCS1, SP100, STAT1, TAP1, TMPRSS4, TNFSF10, TRIM25, UBD, UBR4, XAF1,ZFHX3, AGAP2, AKR1C3, ARG2, ARL6IP5, BCL11A, BCL11B, BCL2, BMPR1B, CALB2, CALCRL, CALR, CAP2, CCR1,CDC42, CFTR, CGA, CPOX, CXCL14, CYP3A5, DBF4, DHFR, DYX1C1, EHHADH, EPHX2, FANCF, FGL1, FLCN,FLG, GLRX, GRK5, GSTA3, HDAC4, HIST2H2BE, HSPA14, HSPD1, KHK, KLHL7, KLK2, KLK8, KRT13, KRT15,LCK, MAPRE2, MBP, MINA, MNT, MYH14, NEK4, NQO2, NUMB, OAZ1, PARD6A, PIGF, PLA1A, POLH, PPP2R2B,PSMD10, PSPH, PTPN1, RAN, RAP1GDS1, RNASE1, SOAT1, SPANXA1, TACC2, TACR3, TARP, TCEA1, TSN,UGT2B7, USHBP1* |
| Breast Cancer | | *ABCA1, B2M, BMP7, BTG2, C1orf38, CAPN1, CEACAM1, CP, DDX58, EIF2AK2, ETV4, ETV7, HSP90B1,HSPA5, ICAM1, IFI27, IL15, IRF1, IRF7, IRF9, ISG15, LAMP3, LGALS3BP, LMO2, MAP3K5, MAPK3, MLL,MUC5AC, MX1, PML, RARRES3, S100A9, SERPINA3, SMG1, SMPD3, SOCS1, SP100, STAT1, TAP1, TNFSF10, TRIM25, UBE2L6, ZFHX3, AKR1C3, BBS9, BCL2, BMPR1B, C11orf17, CALR, CCR1, CCT5, CDC42, CFTR, CGA, CPOX, CUL4A, CXCL14,CYP3A5, CYP4Z2P, DBF4, DHFR, DYX1C1, EPHX2, FANCF, FLCN, FMNL3, GLRX, GSTA3, HIST2H2BE, HSPA14, HSPD1, KLK2, KRT13, KRT15, LCK, MAP2K6, MBP, MNT, MYH14, NEK4, NQO2, NUMB, PARD6A, PPP2R2B,PTPN1, RAN, RNASE1, SOAT1, SPANXA1, SRI, TACC2, TARP, UGT2B7, WIPF1* |
| Metastasis | | *B2M, BMP7, BST2, C3, CEACAM1, CGN, CP, CX3CL1, ETV4, ETV7, FAM3C, HLA-B, HSP90B1, HSPA5,ICAM1, IFI27, IL15, IRF1, LAMP3, LAP3, LGALS3BP, LMO2, MAP3K5, MAPK3, MLL, MUC5AC, MX1, PDIA3, PML, PSMB10, PSMB8, PSMB9, S100A9, SERPINA3, SMG1, SOCS1, STAT1, TAP1, TMPRSS4, TNFSF10, BBS9, BCL2, CALB2, CALR, CCR1, CDC42, CGA, CPOX, CXCL14, CXXC4, DHFR, EIF2C3, FMNL3, GPR68, HSPD1, KLK2, KLK8, KRT13, KRT15, LCK, MAP2K6, MBP, MINA, MYH14, NOL7, NUDT6, PSMD10, RAN, RNASE1, RNF125, SIAH2, SOAT1, ST3GAL4, TPM3* |
| Tumor Progression | | *ABCA1, BMP7, CAPN1, CEACAM1, EIF2AK2, ETV4, ETV7, HSP90B1, HSPA5, ICAM1, IFI27, IL15, IRF1, LAMP3, LGALS3BP, LMO2, MAPK3, MUC5AC, PML, POLQ, S100A9, SERPINA3, SMG1, SOCS1, STAT1,TMPRSS4, TNFSF10, TRIM25, XAF1, ADIPOQ, ATM, CA6, CBL, CYP19A1, DRD2, EDA, FABP3, GLYATL1, GPLD1, GRIN2B, HLA-G, IL10, MAP2, MAPK8, MAX, MYO18B, OCA2, PRKAA2, PRND, PSMD4, SERPINB5, TERF2IP, TTF2, TWIST1* |
| Solid Tumors | | *B2M, BST2, CEACAM1, CP, ETV4, ETV7, HSP90B1, HSPA5, ICAM1, IFI27, IFIT3, IL15, IRF1, LMO2, MLL,MLL4, MUC5AC, PML, SMG1, SOCS1, STAT1, TNFSF10* |
| Lymph Node Metastasis | | *ADAM28, ATP2A2, BRAF, CCND2, CCNL1, CEACAM1, EGFR, ERAP1, ERBB3, ERBB4, FOXP3,HOXC6, MAPK7, MAPRE1, MGAT3, PTK2B, S100A1, SERPINB5, TGFB1, TP73, TRIM25, TSHR,VWF* |
| Oncogenesis | | *AKT3, BRAF, CCND2, CSNK2A1, EGFR, ERBB3, ERBB4, ERG, FOXP3, MAPK7, MLL, NF1, NSD1, PTK2B, REN, SERPINB5, TAP2, TGFB1, TP73, TSHR* |

Each of the ontologies is related to a localization, cellular process or disease and includes a subset of statistically up/down-regulated genes.
